# Influence of Cupric (Cu^2+^) Ions on the Iron Oxidation Mechanism by DNA-Binding Protein from Starved Cells (Dps) from *Marinobacter nauticus*

**DOI:** 10.3390/ijms241210256

**Published:** 2023-06-17

**Authors:** João P. L. Guerra, Daniela Penas, Pedro Tavares, Alice S. Pereira

**Affiliations:** 1 UCIBIO—Applied Molecular Biosciences Unit, Department of Chemistry, NOVA School of Science and Technology, Universidade NOVA de Lisboa, 2829-516 Caparica, Portugal; jp.guerra@campus.fct.unl.pt (J.P.L.G.); danipenass@gmail.com (D.P.); 2 Associate Laboratory i4HB—Institute for Health and Bioeconomy, NOVA School of Science and Technology, Universidade NOVA de Lisboa, 2829-516 Caparica, Portugal

**Keywords:** DNA-binding protein from starved cells (Dps), iron oxidation, ferroxidase centers, iron homeostasis, transition metals in biology, copper in biology, electron paramagnetic resonance spectroscopy (EPR), Mössbauer spectroscopy

## Abstract

Dps proteins (DNA-binding proteins from starved cells) are multifunctional stress defense proteins from the Ferritin family expressed in Prokarya during starvation and/or acute oxidative stress. Besides shielding bacterial DNA through binding and condensation, Dps proteins protect the cell from reactive oxygen species by oxidizing and storing ferrous ions within their cavity, using either hydrogen peroxide or molecular oxygen as the co-substrate, thus reducing the toxic effects of Fenton reactions. Interestingly, the interaction between Dps and transition metals (other than iron) is a known but relatively uncharacterized phenomenon. The impact of non-iron metals on the structure and function of Dps proteins is a current topic of research. This work focuses on the interaction between the Dps from *Marinobacter nauticus* (a marine facultative anaerobe bacterium capable of degrading petroleum hydrocarbons) and the cupric ion (Cu^2+^), one of the transition metals of greater biological relevance. Results obtained using electron paramagnetic resonance (EPR), Mössbauer and UV/Visible spectroscopies revealed that Cu^2+^ ions bind to specific binding sites in Dps, exerting a rate-enhancing effect on the ferroxidation reaction in the presence of molecular oxygen and directly oxidizing ferrous ions when no other co-substrate is present, in a yet uncharacterized redox reaction. This prompts additional research on the catalytic properties of Dps proteins.

## 1. Introduction

Transition metals, while kept at controlled low concentrations, are essential to every organism. Both iron and copper are part of the most abundant intracellular metals as these are incorporated as co-factors of key proteins in respiration, oxygen transport or detoxification mechanisms [1]. However, in excess, Fe or Cu ions become toxic due to their propensity to generate reactive oxygen species (ROS), such as the hydroxyl or superoxide anion radicals, through Fenton and Haber–Weiss reactions [2]. Iron also precipitates as ferric hydroxide at physiological pH. Proper iron homeostasis in bacteria is thus both a challenge and a necessity. 

The members of the Ferritin family of proteins are the most notable intracellular agents of iron storage and management [3]. In general terms, these proteins perform the oxidation and mineralization of ferrous ions into a non-toxic ferric oxide mineral core lodged within their hollow spherical structure [4,5]. The Ferritin family of proteins can be grouped into maxi-ferritins (assemblies of 24 subunits) and mini-ferritins (also termed DNA-binding protein from starved cells or Dps), which are composed of 12 subunits [6]. Besides the activity of iron oxidation and mineralization common to other ferritins, most Dps proteins also protect the cell through direct binding and condensation of bacterial DNA [7]. The importance and morphology of the Dps-DNA condensates have been the focus of intensive research [8,9,10]. Recently it was reported that Dps proteins can undergo spontaneous complex coacervation with different forms of DNA, being responsible for the compaction of the nucleoid in the stationary phase [11], and may also act as a molecular chaperone under heat stress [12], further expanding the protective functions of these proteins. Contrary to maxi-ferritins, Dps proteins are exclusively present in Prokarya. An example is *Marinobacter (M.) nauticus* (formerly known as *M. hydrocarbonoclasticus* [13]), a facultative anaerobe member of the Proteobacteria that produces a Dps protein [14]. This organism has gathered biotechnological significance due to its ability to sustain different levels of oxygen and to degrade non-cyclic hydrocarbons such as those found in oil spills [15,16].

Structurally, Dps proteins present a hollow spherical cube assembly of twelve identical subunits with a highly conserved four-helix bundle motif and N- and C-terminal regions that are mostly unordered [17]. At the interface between each pair of antisymmetric subunits lie the ferroxidase centers (FOCs)—two binding sites per dimer, twelve in total, where the ferroxidation reaction occurs, making the dimer the functional unit of the protein [18]. The amino acid residues responsible for the coordination of the ferrous ions at the FOC are highly conserved throughout the Dps family and usually consist of two histidine and one aspartate/glutamate residues from one subunit and two aspartate/glutamate residues from the opposing subunit [6]. After ferrous iron binding, the subsequent ferroxidation reaction (oxidation of Fe^2+^ to Fe^3+^) is coupled to the reduction of a co-substrate, either H_2_O_2_ or O_2_ [19,20]. In contrast to maxi-ferritins, Dps proteins prefer H_2_O_2_ as the oxidant, as the ferroxidation rate is generally 100-fold faster (occurring in the millisecond range timescale) when compared to the reaction with molecular oxygen (occurring within the seconds-to-minutes timeframe) [21]. Recently, an alternative mechanism of self-catalytic formation of the mineral in the absence of either co-substrate was also described [14]. Oxidized ferric species are then translocated through intra-protein channels before reaching nucleation sites in the internal surface of the protein, where the ferrihydrite mineral core is formed [22,23]. Up to 500 iron ions can be stored per Dps protein molecule [24]. 

Besides Fe, Dps proteins are also able to bind to other transition metals [6,25,26]. Mn^2+^, Zn^2+^, Co^2+^ and Cu^2+^ have been found bound to the FOC with moderate affinity in different Dps homologs [27,28,29,30]. Interestingly, previous studies have shown that these metals can affect the activity of the protein and its iron mineralization mechanisms. For example, the binding of Mn^2+^ and Zn^2+^ ions had a rate-enhancing effect in the overall O_2_-driven iron uptake mechanism in Dps proteins from *Kineococcus radiotolerans* and *Thermosynechococcus elongatus*, respectively [27,31]. In other cases, such as the Dps homologs from *Listeria innocua*, *Streptococcus (S.) suis* or *Nostoc punctiforme*, Zn^2+^ and Tb^3+^ binding displace the iron from the FOCs, inhibiting the ferroxidation reaction [32,33,34]. 

So far, the binding of Cu^2+^ ions to the FOCs has only been reported for the Dps from *S. suis* [30], with its copper-bound atomic structure revealing the FOCs occupied with Cu^2+^ in a coordination similar to the Fe^2+^ ions. Notwithstanding, Cu^2+^ features as an important co-factor in biological systems, with copper centers being present in several enzymes involved in electron transfer, free radical scavenging or energy production [35].

**Figure 1:**
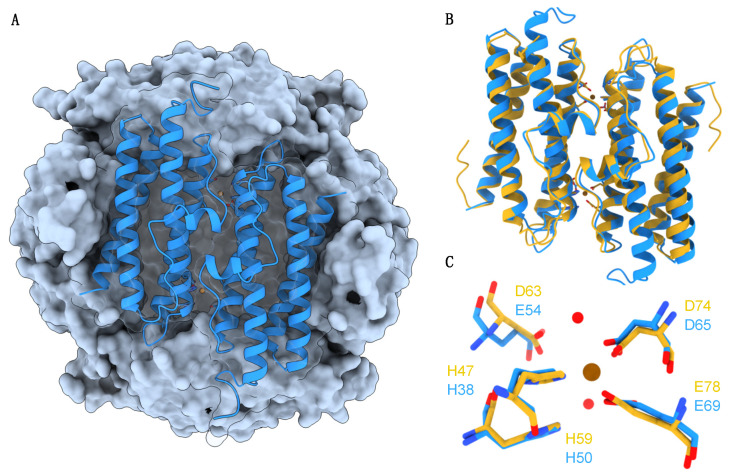
Structural illustration of the Dps from *M. nauticus*. (**A**) Macromolecular structural model of the Dps cube-like dodecamer assembly (light blue surface) with one of its facets (dimer) shown as blue ribbons. (**B**) The antisymmetric dimer of *M. nauticus* Dps (in blue) and *Streptococcus suis* Dpr (in yellow). The residues that compose the two ferroxidase centers (FOCs) formed at the dimer interface are shown as sticks, together with brown spheres representing Cu^2+^ ions. (**C**) Magnification of the residues that create the FOCs in *M. nauticus* Dps (carbon atoms in blue) and Dpr (carbon atoms in yellow). The Cu^2+^ ion is shown as a brown sphere. Structural waters are presented as red spheres. The *M. nauticus* Dps model was obtained using SWISS-MODEL [36] as a structure-homology modeling tool and the atomic structure of *S. suis* Dpr bound to Cu^2+^ ions as template (PDB ID 2XJN).

In this work, we aimed to examine the effect of Cu^2+^ ions on the structure and function of *M. nauticus* Dps. This Dps homolog exhibits high structural homology with *S. suis* Dpr (a member of the Dps family), including a putatively identical FOC coordination site (Figure 1). Further probing if Cu^2+^ ions bind to Dps, we intended to determine the impact of Cu^2+^-binding on the iron oxidation mechanism in both anaerobic and aerobic conditions, mimicking the different environments that the bacterium *M. nauticus* thrives in, using spectroscopic techniques (electron paramagnetic resonance, Mössbauer and UV/Visible spectroscopies).

## 2. Results

### 2.1. Binding of Cupric Ions to Dps

**Figure 2:**
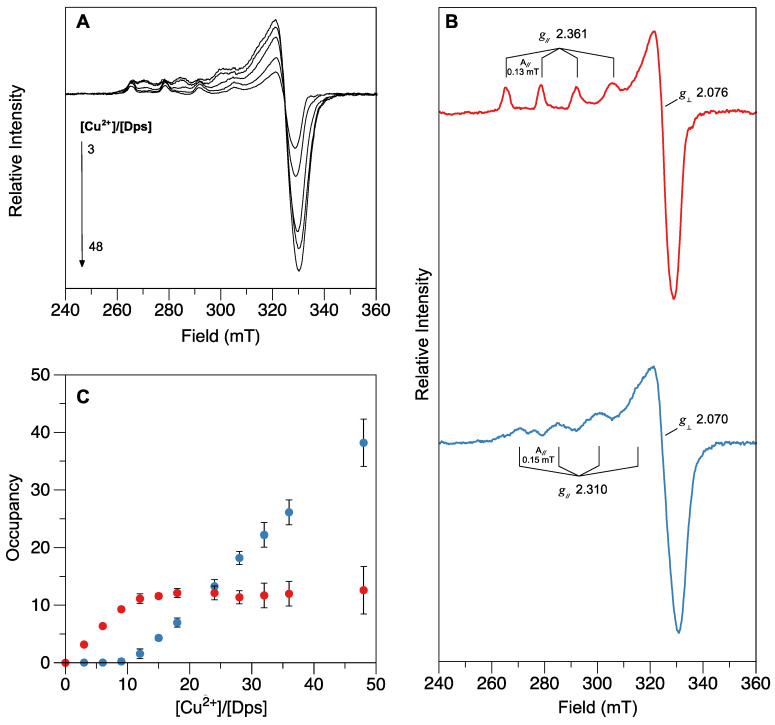
Spectroscopic characterization of the Cu^2+^ binding sites in Dps by EPR spectroscopy. (**A**) X-band spectra at 77 K of Dps samples incubated with 3 to 48 eqs of Cu^2+^ per Dps dodecamer. Other experimental conditions: microwave frequency, 9.45 MHz; microwave power, 20.0 mW; modulation amplitude, 0.1 mT; receiver gain, 5 × 10^2^. (**B**) Deconvolution of the spectra shown in (**A**) into two Cu signals (Site I in red and Site II in blue). (**C**) Contribution of each Cu signal in each spectrum obtained by titrating the protein with Cu^2+^ (up to 48 Cu^2+^/Dps) after spectral deconvolution (Site I in red and Site II in blue). Occupancy of each binding site is defined herein as the molar ratio of cupric ions per Dps dodecamer.

Electron paramagnetic resonance (EPR) spectroscopy is often used to probe metal-binding sites in metalloproteins, provided the metal is in a paramagnetic oxidation state, such as Cu^2+^ ions. The spectra of Dps samples incubated with different amounts of Cu^2+^ (between 3 and 48 Cu^2+^/Dps dodecamer), presented in Figure 2A, display axial EPR signals with parallel and perpendicular features characteristic of Type II copper centers (T2Cu), typically resulting from square planar coordination by N or N/O ligands. Samples reacted with 3 to 12 Cu^2+^/Dps give EPR spectra with a single EPR signal, here termed Site I, with $g_{∥}$ = 2.361, $A_{∥}$ = 0.13 mT and $g_{⊥}$ = 2.076 (Figure 2B, in red). With the addition of more than 12 Cu^2+^/Dps, EPR spectra explained with two sets of EPR parameters were detected. Spectral deconvolution (using Site I as reference spectrum) indicates that those can be described as a sum of Site I and a novel Site II signal, with spectroscopic parameters as follows: $g_{∥}$ = 2.310, $A_{∥}$ = 0.15 mT and $g_{⊥}$ = 2.070 (Figure 2B, in blue). Both signals are, therefore, characterized by axial symmetry with copper hyperfine splitting in the parallel region. By global deconvolution of all titration spectra and calculation of the occupancy of each type of Cu^2+^ signal (Figure 2C), one can conclude that Site I exhibits a higher affinity towards Cu^2+^ ions, saturating at 12 Cu^2+^/Dps. This can be interpreted as the occupation of the FOC binding site present in each protein subunit (in a total of 12 FOCs per protein dodecamer), constituted by amino acid side chains compatible with T2Cu EPR signals (histidine, glutamate and aspartate). Furthermore, the occupation of Site II follows a sigmoidal profile, being occupied only after Site I becomes saturated (from 12 up to 48 Cu^2+^/Dps), which is consistent with the occupation of a second, weaker binding site. The Site II signal is also less defined and broader, which may be explained by Cu^2+^ ions binding to the electronegative regions in the tri-fold pores and/or intra-protein channels. As such, the EPR data suggest that the Cu^2+^ ions bind in a sequential manner to at least two distinct sites, the first producing a highly stable species (most likely resulting from the binding of Cu to the FOCs).

### 2.2. Effect of Cu^2+^-Binding on the Iron Mineralization Rate by Dps

**Figure 3:**
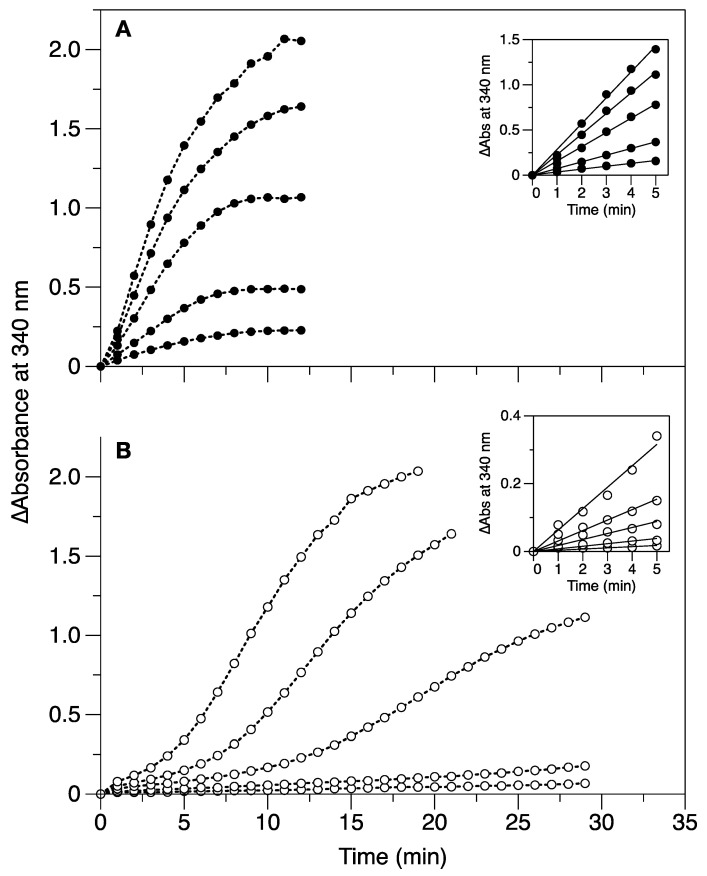
Monitoring the iron oxidation reaction by apo- and 12 Cu^2+^-loaded Dps. Kinetic profiles obtained upon addition of different amounts of Fe^2+^/Dps in 200 mM MOPS pH 7.0, 200 mM NaCl in atmospheric oxygen conditions. Addition of 48 to 384 Fe/Dps (from bottom to top traces) to (**A**) Dps samples pre-loaded with 12 Cu^2+^ eqs per dodecamer and (**B**) apo-Dps samples. The insets are magnifications of the first 5 data points used to determine the initial velocities as per Table 1. A representative result of three independent assays is shown. Dotted lines were included as visual aids to data trends.

The iron oxidation and mineralization activity rates of apo-Dps and Cu^2+^-bound Dps (12 Cu^2+^ per Dps dodecamer) were compared using UV/visible spectrophotometry. The iron loading assays (from 48 to 384 Fe^2+^/Dps) were performed in atmospheric oxygen conditions to generate ferric species with a brownish color, characterized by a broad absorption band between 300 and 400 nm. The kinetic profiles obtained monitoring the absorbance at 340 nm after each iron addition (Figure 3) illustrate the effect of cupric ion binding on the iron oxidation mechanism catalyzed by Dps. Whilst the apo-Dps kinetic curves (Figure 3B) present the sigmoidal shape typical of the iron/O_2_ uptake reaction by Dps proteins, the curves of the 12 Cu^2+^-Dps samples (Figure 3A) show a pseudo-first-order kinetic behavior, with a much faster iron oxidation reaction rate. In the former, the initial slower phase shortens with increasing Fe^2+^ substrate concentration added to the protein due to the major contribution of the auto-catalytic iron oxidation activity of the mineral core built up inside the protein cavity.

The initial velocity, *v*_0_ as ∆Abs/min, of the iron oxidation reaction was determined by computing the slope of the tangent to the inception (*t* = 0) of each progress curve (see insets in Figure 3). The results obtained are presented in Table 1 and show up to ~10-fold increase on *v*_0_ when Dps was pre-loaded with 12 Cu^2+^/dodecamer (e.g., from 0.015 ∆Abs/min to 0.161 ∆Abs/min for 192 Fe/Dps). Overall, the results from this experiment demonstrate the functional impact of cupric ion binding to Dps—the initial rate of the iron oxidation reaction (ferroxidation and mineralization) in the presence of molecular oxygen (the least preferred co-substrate) is significantly increased, suggesting that Cu^2+^ participates in the redox chemistry steps of iron oxidation.

### 2.3. Characterization of the Anaerobic Iron Uptake Reaction in the Presence of Cu^2+^ Ions

**Figure 4:**
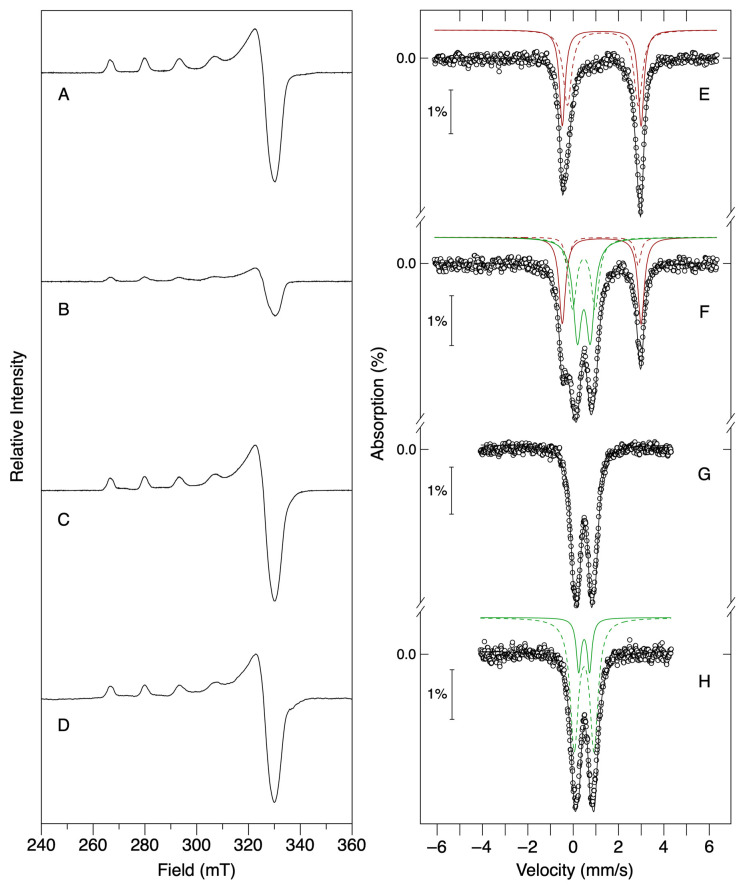
Spectroscopic characterization of Dps samples probing the redox reaction between Cu^2+^ and Fe^2+^ ions within the protein. Left Panel: EPR spectra recorded at 77 K. Other experimental conditions: microwave frequency, 9.45 MHz; microwave power, 20.0 mW; modulation amplitude, 0.1 mT; receiver gain, 5 × 10^2^. Right panel: Mössbauer spectra recorded at 77 K in the absence of an external applied field. (**A**) Cu^2+^-loaded Dps sample (12 eqs. of Cu^2+^ per Dps dodecamer); (**B**,**F**) Cu^2+^-loaded Dps sample, after reaction with 12 eqs of Fe^2+^ for 20 min in anaerobic conditions. (**C**,**G**) Addition of a 2-fold molar excess of H_2_O_2_ to the 12 Cu^2+^-loaded Dps sample after incubation with 12 Fe^2+^ per dodecamer. (**D**) Addition of 12 Fe^2+^ to the 12 Cu^2+^-loaded protein sample in atmospheric oxygen conditions. (**E**) Dps incubated with 12 eqs of Fe^2+^ per dodecamer; (**H**) Dps incubated with 12 eqs of Fe^2+^ and 2-fold molar excess of H_2_O_2_. The solid lines overlaying the experimental spectra are the result of least squares fits to the data (relevant parameters listed in Table 2). Contributions of ferrous species shown in red (species I and II in solid and dashed lines, respectively) and ferric species shown in green (species III and IV in solid and dashed lines).

To better understand the effect observed in the Cu^2+^-loaded Dps, protein samples of Dps pre-loaded with 12 Cu^2+^ eqs per dodecamer were incubated for 20 min with 12 Fe^2+^ eqs (1 Cu^2+^/1 Fe^2+^/FOC) in anaerobic conditions, without any additional co-substrate. Parallel samples were analyzed using EPR and Mössbauer spectroscopies (Figure 4 and Table 2).

The EPR spectrum of the initial protein sample, Cu^2+^-loaded Dps (Figure 4A), shows the previously observed characteristic axial EPR Cu^2+^ signal (as in Figure 2B). In turn, incubation of apo-Dps with 12 Fe^2+^/dodecamer (Figure 4E) generates a Mössbauer spectrum explained by the contributions of high-spin ferrous species (isomer shift, *δ* = 1.26 and 1.31 mm/s and quadrupole splitting, ∆*E*_Q_ = 3.48 and 3.12 mm/s), as described in previous works [14,37].

Analysis of the spectra obtained after the addition of 12 Fe^2+^ eqs to 12 Cu^2+^-Dps samples (Figure 4B,F) suggests that the Cu^2+^ ions are directly responsible for oxidizing ferrous ions in a 1:1 redox reaction. In this case, the EPR spectrum reflects an identically shaped signal with a 65% loss in spin concentration, whilst the Mössbauer spectrum can be described as the sum of 34% of high-spin ferrous species (red sub-spectrum) and 66% of a new sub-spectrum with parameters characteristic of high-spin ferric species with oxygen/nitrogen ligands (*δ* = 0.47 mm/s, ∆*E*_Q_ = 0.57, 1.00 mm/s). Together, these results can be interpreted as evidence for the concurrent oxidation of Fe^2+^ to Fe^3+^ coupled with the reduction of Cu^2+^ to Cu^+^, with the latter being diamagnetic and thus EPR silent. Please note that in these anoxic experimental conditions, no other oxidant species exist besides the cupric ions.

### 2.4. The Initial Cu^2+^ State of Copper Is Regenerated in the Presence of an Oxidant Co-Substrate

Since copper and iron co-occur in biotic environments, and considering the facultative anaerobe nature of *M. nauticus* (as well as other known Dps-expressing organisms) and its ability to degrade different types of hydrocarbons (including petroleum hydrocarbons), it is important to investigate the potential synergetic effect of both metals. The Mössbauer and EPR data shown above indicate that copper directly oxidizes Fe^2+^ ions in anaerobic conditions accepting one electron in a stoichiometric redox reaction. Additionally, the kinetic progress curves of 12 Cu^2+^-Dps samples reacted with Fe^2+^ ions (48 to 384 Fe/Dps) in atmospheric oxygen conditions demonstrate that the rate-enhancing effect is quantitative, indicating a catalytic role for copper ions. As such, and to clarify the oxidation state of both copper and iron ions during the aerobic iron oxidation reaction, parallel EPR and Mössbauer samples were prepared in different conditions (Figure 4). As discussed in the previous section, the anaerobic incubation of the 12 Cu^2+^-Dps sample with ferrous ions leads to the loss of the Cu^2+^-Dps EPR signal with the concomitant stoichiometric appearance of ferric species in the Mössbauer spectrum. However, if such incubation is conducted under aerobic conditions, no loss of the EPR signal is observed (Figure 4D). Furthermore, the addition of an excess of H_2_O_2_ after sequential incubation of 12 Cu^2+^-Dps with 12 Fe^2+^ in anaerobic conditions for 20 minutes (i.e., addition of an oxidative co-substrate after the 1:1 reaction shown in Figure 4B,F) results in the detection of an EPR signal corresponding to a fully oxidized 12 Cu^2+^-Dps sample as well as to the presence of 100% ferric species, as judged by Mössbauer spectroscopy (Figure 4C,G). Interestingly, the parameters obtained for the ferric species generated by the reaction with Cu^2+^ are consistent with the ferric parameters obtained after co-incubating Dps with 12 eqs of Fe^2+^ with a two-fold molar excess of H_2_O_2_ (Figure 4H) and with previous reports of the ferric mineral obtained using these co-substrates of the Dps iron mineralization reaction [14], indicating that the ferric product of the Cu^2+^:Fe^2+^ redox reaction in anaerobic conditions most likely shares a similar chemical composition/structure.

Altogether, our results point towards the cupric ion exerting a significant rate-enhancing effect on the catalytic activity of Dps, when compared with the reaction using molecular oxygen as a co-substrate, directly coupling the oxidation of ferrous ions with its reduction to Cu^+^. The observation that Cu^+^ can be re-oxidized to Cu^2+^ by either O_2_ or H_2_O_2_ further supports the catalytic role of copper ions in ferrous iron oxidation and concomitant co-substrate reduction.

## 3. Discussion

The bimodal cell protective effect accomplished by Dps proteins through ferrous iron scavenging and DNA shielding has been studied for over two decades. In general, the Dps catalytic activity (ferroxidation and mineralization) using H_2_O_2_ (preferably) or O_2_ co-substrate has been characterized since their discovery, with an alternative self-catalytic mineral core growth mechanism in the absence of any co-substrate being only recently described.

In this work, the functional properties of the Dps from *M. nauticus* were further explored whilst probing the interplay between the protein, iron, and cupric ions. Based on previous data from the literature, we postulated the hypothesis that the occupation of the Dps ferroxidase centers by non-iron biologically relevant transition metals, previously detected using several methods (spectroscopic, calorimetry methods or structure determination), arise from a biological advantage and do not only represent a putative metal binding capacity of the FOCs.

Copper complexes have been extensively studied using multiple spectroscopic techniques, specifically EPR, X-ray absorption and UV/Visible spectroscopies, among others [38,39,40]. In this study, EPR and Mössbauer spectroscopies were applied to detect and quantify the Cu-/Fe-containing species, as both techniques have proven to be important and complementary tools to characterize metalloproteins. While EPR active copper centers present characteristic EPR spectra (for example, type II centers show axial signals with hyperfine splitting in the parallel region), Mössbauer spectroscopy allows the identification of all iron species, regardless of their oxidation state and coordination geometry [41].

The Cu^2+^ titration experiment performed in this work using EPR spectroscopy (Figure 2) yielded the first direct evidence of the binding of cupric ions to the FOCs of a Dps protein apart from crystallographic information. The EPR spectra of Dps samples incubated with up to 12 eqs of Cu^2+^ per dodecamer show a single well defined axial signal characteristic of Type II Cu centers. The relatively high sharpness of this signal suggests that the chemical environment around the Cu^2+^ ions is homogenous, with a coordination sphere of N/O ligands, such as residues His38, His50, Glu54, Asp65 and Glu69 that constitute the FOC in the 12 subunits of the *M. nauticus* Dps dodecamer (as highlighted in Figure 1), in agreement with previous crystallographic evidence of such Cu^2+^-FOC centers in *S. suis* Dpr. After what we believe to be the saturation of the FOC binding sites, the Cu^2+^ ions most likely start to bind to other sites scattered along the N-terminal pores and/or intra-protein channels, resulting in a second, subsequent Type II Cu EPR broader signal, suggesting different coordination geometries and affinities. Such a type of binding is commonly observed in crystallographic structures of metal–Dps complexes [42,43].

Here, we described, for the first time, the rate-enhancing effect of Cu^2+^ binding to *M. nauticus* Dps in the iron uptake reaction, which resulted in up to ~10-fold faster initial velocity of iron oxidation in the presence of O_2_ (data presented in Figure 3 and Table 1). The impact of non-iron metal binding on the catalytic properties of the protein was described for other metal ions such as Zn^2+^, Mn^2+^ and Tb^3+^ but not Cu^2+^. In the cases of interactions of Zn^2+^ ions with the cyanobacterium *Thermosynechococcus elongatus* Dps A [31] and Mn^2+^ with the radiation-resistant bacterium *Kineococcus radiotolerans* Dps [27], the presence of these metal cations resulted in an iron oxidation rate-enhancing effect, especially in the presence of O_2_, with a ~20-fold faster rate (compared with apo-Dps incubated with iron) in the former. Strikingly, partial or complete inhibition of the iron oxidation reaction using spectrophotometry was detected when *Listeria innocua* Dps [32] and *Nostoc punctiforme* Dps4 [34] were previously incubated with Zn^2+^ and/or Tb^3+^, suggesting that the catalytic properties of metal–Dps complexes may depend on the type of divalent metal and/or specific protein residues.

Furthermore, this work probed a putative redox reaction between Cu^2+^ and Fe^2+^ ions in Dps by spectroscopically characterizing the iron oxidation reaction in anaerobic conditions with no additional co-substrate. Our results show that in these conditions, Cu^2+^ and Fe^2+^ participate in a 1:1 electron transfer process that concurrently generates oxidized Fe^3+^ species and reduced Cu^+^ ions (Figure 4 and Table 2). The latter may be able to reduce a substrate molecule, regenerating the initial Cu^2+^ state and thus allowing the next Cu–Fe redox turnover. In general terms, the 1:1 reaction detected in anaerobic conditions is most likely the mechanism behind the rate-enhancing effect detected in atmospheric oxygen conditions using spectrophotometry. The rate-enhancing catalytic effect of Cu^2+^ ions is independent of the amount of iron added since the reaction should now be controlled by the rate of Cu^2+^–Cu^+^ cycling.

Overall, the data presented in this work reveal a yet uncharacterized catalytic synergetic effect between iron and copper in the iron oxidation reaction by Dps, one of the two primary functions of these stress response proteins. The effect of Cu^2+^ ions on Dps catalysis may represent a response mechanism to multiple stressors in the presence of O_2_. Under these conditions, Dps shows atypical reactivity sequestering the ferrous iron more efficiently and decreasing cellular exposure to ROS formed by Fenton chemistry (since the rate of iron oxidation is significantly greater than the rate of the apo-form using O_2_ as co-substrate). Moreover, the ability of Cu^2+^-loaded Dps to directly oxidize Fe^2+^ ions in anoxic conditions without any additional co-substrate is of utmost interest and prompts additional research into the mechanistic properties of Dps catalysis in facultative aerobic bacteria such as *M. nauticus* and the possible significance of non-iron metal ions as regulators of the Dps function.

## 4. Materials and Methods

### 4.1. Production of M. nauticus Dps

The production and purification of *M. nauticus* Dps were carried out following the protocols described by Penas et al. [14].

### 4.2. Cu^2+^ Binding Assays by EPR Spectroscopy

A Cu^2+^ stock solution was prepared using >99% pure CuSO_4_⋅5H_2_O (Sigma-Aldrich, now MERCK, Darmstadt, Germany) and Milli-Q water at pH 2.0 and calibrated through the Zincon colorimetric assay [44]. Binding of cupric ions to Dps was monitored by X-band continuous-wave electron paramagnetic resonance (CW-EPR). Triplicate samples of 5–10 µM Dps (dodecamer) in 200 mM MOPS pH 6.0 and 200 mM NaCl buffer were supplemented with increasing concentrations of CuSO_4_ up to a molar ratio of 48 Cu^2+^/Dps and incubated for 10 min at room temperature. Samples were then transferred to 2 mm quartz EPR tubes (Wilmad, ATS Life Sciences Scientific Products, Warminster, PA, USA), flash-frozen in liquid nitrogen, and the spectra were acquired in a Miniscope MS400 spectrometer (Magnettech, now Bruker, Billerica, MA, USA) equipped with a cold finger. Spin quantitations (and corresponding Cu^2+^ concentrations) were calculated by double integration of signals obtained under non-saturating conditions and comparison with a Cu(II)-EDTA standard [45]. Occupancy of each binding site is defined herein as the molar ratio of cupric ions per Dps dodecamer. The data presented in Figure 2 correspond to the average value of the deconvolution of three independent assays, with error bars representing 95% confidence intervals.

### 4.3. Iron Uptake Kinetic Assays

Dps samples were prepared at a concentration of 0.6 µM (7.2 µM monomer) in the apo-form or pre-loaded with 12 Cu^2+^ ions per protein (7.2 µM CuSO_4_) in 200 mM MOPS pH 7.0, 200 mM NaCl buffer. The absorption spectra, between 250 and 800 nm, were registered using an Evo 300 UV/Vis Spectrophotometer (Thermo Fischer Scientific, Waltham, MA, USA) in a 1 cm pathlength quartz cell (Hellma GmbH & Co. KG, Müllheim, Germany) with constant stirring at room temperature. Then, different concentrations of Fe^2+^ ions were added (between 48 and 384 Fe/Dps corresponding to 28 to 230 µM FeSO_4_), initiating the reaction. Absorbance spectra were then acquired in 1 min steps, and the absorbance at 340 nm was plotted over time after baseline correction. Ferrous sulfate stock solutions were freshly prepared, and Fe^2+^ concentration was determined using the 1,10-phenantroline colorimetric assay, as described in [14]. The data presented in Figure 3 are representative of three independent kinetic assays. The initial velocity, *v*_0_ as ∆Abs/min, of the iron oxidation reaction was determined by computing the slope of the tangent to the inception (*t* = 0) of each progress curve, along with estimation of the standard error of the fit with a 95% confidence interval.

### 4.4. EPR and Mössbauer Spectroscopies Characterization

Mössbauer and EPR samples in anaerobic conditions were prepared inside an anaerobic chamber (<4 ppm O_2_; MBLab, MBraun, Garching, Germany). For Mössbauer analysis, a ^57^FeSO_4_ stock solution was prepared by dissolution of a ^57^Fe metal foil with 1 M H_2_SO_4_ followed by quantification of the Fe^2+^ concentration using the 1,10-phenantroline colorimetric assay [14]. Apo-Dps samples at 90 µM in 200 mM MOPS pH 7.0, 200 mM NaCl buffer were incubated anaerobically with 1.08 mM CuSO_4_ (12 Cu/Dps molar ratio) for 20 min at room temperature followed by the addition of 1.08 mM ^57^FeSO_4_ (12 Fe/Dps and 1 Fe/Cu) and left to react for another 20 min at room temperature. Then, 400 μL were transferred to a Mössbauer cell and flash-frozen in liquid nitrogen. At this stage, 2.16 mM of hydrogen peroxide was added (2-fold molar excess over iron) to a parallel sample and further incubated for 20 min before flash-freezing. Mössbauer spectra were recorded on a weak-field spectrometer operating in a constant acceleration mode in transmission geometry, equipped with a Janis Closed Cycle He gas refrigerator cryostat with temperature set at 77 K. The zero velocity of the Mössbauer spectrum is referred to as the centroid of the room-temperature spectrum of a metallic iron foil. Mössbauer analysis was conducted using the WMOSS program (SEE Co., Minneapolis, MN, USA). Parameters (isomer shift, *δ*, quadrupole splitting, ∆*E*_Q_ and linewidths), average values, relative percentages and corresponding uncertainties of each iron species in each sample were obtained by spectral deconvolution through least-square fitting using WMOSS. EPR spectra were collected and analyzed as previously described. For the EPR samples subsequently reacted with O_2_, the anaerobic procedure was reproduced outside the glovebox in atmospheric oxygen conditions.

## 5. Conclusions

The multifaceted features of Dps proteins have gathered significant interest in bacterial protein biochemistry and bio-nanotechnology for more than two decades. Some works in recent years focused on the interaction between Dps proteins and non-iron metals of biological relevance. The influence of divalent transition metal ions (such as Zn^2+^, Co^2+^, Cd^2+^) regarding protein structural dynamics has been implicated in protein oligomerization and DNA-binding properties, which further the importance of probing possible roles for metal ions other than the ferrous/ferric ions.

This work pays attention to the hitherto lesser-explored interaction between Dps and cupric ions, one of the most relevant metals in biology, of particular importance in redox biochemistry. Our results further advance the recent thought that Dps proteins possibly use metal co-factors to regulate their activity by showing that Cu^2+^ ions occupy specific sites within the protein and enhance the rate of iron oxidation in aerobic conditions, directly serving as an electron acceptor for the iron oxidation in anoxic conditions with no additional co-substrate. These findings reveal a versatility towards ferrous ion oxidation and storage on par with the stated metabolic versatility of bacteria. In fact, *M. nauticus* can adapt to different environmental conditions. In the total absence of molecular oxygen, this bacterium obtains energy from nitrate respiration; however, in microaerophilic conditions, the oxidative phosphorylation terminal oxidase reduces molecular oxygen to hydrogen peroxide [46,47]. In these conditions, Dps can rapidly process both ferrous ions and hydrogen peroxide, providing protection and storage functionalities. In aerobic conditions, *M. nauticus* will shift to the use of a terminal oxidase capable of fully reducing molecular oxygen to water. Under these conditions, it is fair to assume that, with cupric ions available to bind to Dps, molecular oxygen would be more efficiently used as a co-substrate in the ferrous oxidation and storage reaction.

## Figures and Tables

**Table 1:** Comparison of the initial velocity of iron oxidation between apo-Dps and 12 Cu^2+^-Dps samples upon addition of different concentrations of Fe^2+^ ions.

[Fe^2+^]/[Dps]	Initial Velocity (∆Abs/min)	
	Apo-Dps	12 Cu^2+^-Dps
48	0.003 ± 0.001	0.031 ± 0.002
96	0.006 ± 0.002	0.074 ± 0.002
192	0.015 ± 0.003	0.161 ± 0.005
288	0.028 ± 0.003	0.231 ± 0.009
384	0.064 ± 0.004	0.290 ± 0.012

**Table 2:** Mössbauer parameters of the iron species detected during the iron oxidation reaction in the presence of cupric ions by *M. nauticus* Dps.

Iron Species	Mössbauer Parameters			Absorption (%)		
	*δ* (mm/s)	∆*E*_Q_ (mm/s)	Linewidth (mm/s)	12 ^57^Fe^2+^	12 ^57^Fe^2+^ + 12 Cu^2+^	12 ^57^Fe^2+^ + 12 Cu^2+^ + 24 H_2_O_2_
Ferrous species						
I	1.26 ± 0.02	3.48 ± 0.02	0.30 ± 0.02	46 ± 2	22 ± 3	−
II	1.30 ± 0.02	3.12 ± 0.03	0.38 ± 0.03	54 ± 2	13 ± 3	−
Ferric species						
III	0.48 ± 0.02	0.61 ± 0.02	0.38 ± 0.03	−	44 ± 3	62 ± 2
IV	0.48 ± 0.02	1.01 ± 0.02	0.38 ± 0.02	−	21 ± 3	38 ± 2

Note: Contribution of each ferrous and ferric species in each spectrum after deconvolution given as percentages.

## Data Availability

Not applicable.
